# Solanesol: a promising natural product

**DOI:** 10.3389/fphar.2025.1504245

**Published:** 2025-03-24

**Authors:** Yinchao Ma, Ge Wei, Zhichen Dong, Ziyuan Wang, Xinlong Zhai, Yuan Liu, Huan Chen, Yaning Fu, Hongwei Hou, Qingyuan Hu, Ming Chu

**Affiliations:** ^1^ Beijing Life Science Academy, Beijing, China; ^2^ NHC Key Laboratory of Medical Immunology (Peking University), School of Basic Medical Sciences, Peking University, Beijing, China

**Keywords:** solanesol, antimicrobial, antioxidant, anti-inflammatory, membrane stabilization

## Abstract

Solanesol, identified as Nonaprenol alcohol, predominates in the *Solanaceae* family. This compound exists as a white to pale yellow solid at room temperature, characterized by low polarity and water insolubility. Its unique chemical structure—featuring nine non-conjugated double bonds and low polarity—confers remarkable biological activities. Recent studies have demonstrated that solanesol exhibits polypharmacological properties, including antimicrobial, antioxidant, anti-inflammatory, and membrane-stabilizing effects. Mechanistically, solanesol suppresses ROS generation and inhibits pro-inflammatory cytokines (IL-1β, TNF-α). Preclinical studies highlight its therapeutic potential in inflammatory disorders (periodontitis, neuropathic pain) and neurodegenerative diseases (Alzheimer’s, Parkinson’s). However, current research still faces critical bottlenecks, such as a lack of *in vivo* pharmacokinetic data, unclear molecular targets, and insufficient toxicity assessments. Future studies urgently need to integrate experimental approaches, including target screening, nanotechnology-based delivery systems, and multi-omics analysis, to elucidate its mechanisms of action and promote clinical translation. As a compound that combines natural safety with multi-target effects, solanesol is not only a research focus for the development of novel drugs but also a bridge connecting natural products and precision medicine, poised to lead the innovation of next-generation biocompatible therapies.

## 1 Introduction

**FIGURE 1:**
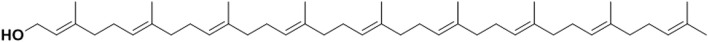
Molecular structure of solanesol (PubChem CID: 5477212).

Solanesol, also known as Nonaprenol alcohol (C_45_H_74_O), is a long-chain unsaturated fatty alcohol belonging to the triterpenoid class (Yan et al., 2019) (Figure 1). Solanesol is primarily found naturally in plants of the *Solanaceae* family, including tobacco, potatoes, tomatoes, eggplants, and peppers. Among these, tobacco contains the highest concentration of solanesol, reaching up to 4.13% of the tobacco’s dry weight (Xiang et al., 2017). The compound was first isolated from tobacco by Rowland and colleagues in 1956 (Rowland et al., 1956). In the early 1980s, Japan achieved the initial successful extraction of solanesol from tobacco leaves. Since the 1990s, China has been extracting solanesol from waste tobacco extracts using methods such as supercritical fluid extraction, ultrasonic-assisted extraction, microwave-assisted extraction, and solvent extraction. The extraction technologies of solanesol have garnered significant attention due to their critical applications in pharmaceutical and industrial fields. Current mainstream methods include: ammonia leaching pretreatment-assisted extraction, dynamic saponification extraction, ultrasonic-assisted extraction, molecular distillation, supercritical CO_2_ fluid extraction, and bio-enzymatic extraction (Yan et al., 2019). Additionally, molecularly imprinted polymers (MIPs) have emerged as a novel purification strategy, enabling selective separation through specific molecular recognition (Long et al., 2015; Ma et al., 2016; Duan et al., 2019). These methods exhibit distinct advantages in efficiency, environmental friendliness, and cost-effectiveness.

Solanesol appears as a white or faintly yellow solid at room temperature, has low polarity, is insoluble in water, and is soluble in hexane and ethanol (Liu et al., 2007). The structure of solanesol, composed of nine isoprene units including nine non-conjugated double bonds, gives it strong biological activity and makes it prone to reactions such as oxidation, addition, and dehydrogenation (Yan et al., 2015). Current research has found that solanesol, as an oil-in-water (O/W) emulsion, has good stability and exhibits comparable physicochemical stability to squalene under storage conditions of 5°C and 25°C. At storage conditions of 40°C, the physical stability of the solanesol emulsion is also good (Fisher et al., 2023). Currently, solanesol is primarily utilized in synthesizing compounds containing the isoprenoid structure, such as coenzyme Q10, vitamin K2, and vitamin E, establishing its indispensable role as an intermediate in ubiquinone-type medications. Recent studies have identified that solanesol can also act as an “active carrier” in the development of novel nano-drug delivery systems for synthesizing anticancer drugs (Gendron et al., 2023). Notably, solanesol inherently exhibits antibacterial, antioxidant, anti-inflammatory, and membrane-stabilizing properties (Table 1) (Zhang et al., 2024), and demonstrates promising therapeutic effects in animal models of inflammatory diseases and central nervous system (CNS) diseases. The exploration of bioactivities and pharmacological effects of natural products derived from plants has recently gained significant momentum. For example, the study by Ullah et al. revealed that nanocomposites based on natural products can exhibit robust antibacterial and anti-biofilm activities through synergistic molecular interactions (Ullah et al., 2024). Therefore, this review summarizes the research progress of solanesol’s polypharmacological actions, aiming to identify critical research gaps and provide a theoretical foundation for further exploration of its medicinal value.

**TABLE 1:** The bioactivity and pharmacological effects of solanesol.

Bioactivity	Effects	Experimental subjects	Dose	References
Antibacterial	Inhibiting the growth of *Staphylococcus aureus, Mycobacterium phlegm, Escherichia coli* and *Pseudomonas aeruginosa*	*Staphylococcus aureus, Mycobacterium phlegm, Escherichia coli* *Pseudomonas aeruginosa*	500 µg/disk 1,000 µg/disk	Chen et al. (2007)
	Inhibition of *Staphylococcus aureus, Bacillus subtilis, Escherichia coli* growth	*Staphylococcus aureus* *Bacillus subtilis* *Escherichia coli*	75 mg/L, 150 mg/L 300 mg/L, 600 mg/L 900 mg/L, 1,125 mg/L 1,500 mg/L, 2,250 mg/L	Guo et al. (2008)
Antioxidant	Scavenging DPPH free radicals	None	100 μg/mL	Huang et al. (2008)
	Scavenging superoxide anion free radical and hydroxyl free radical, inhibiting microsomal lipid peroxidation	Mice	30 mg/kg, 60 mg/kg 120 mg/kg	Ma et al. (2011)
	Absorption of UV radiation, scavenging of lipid free radicals, inhibition of tyrosinase activity	None	10 mg/L, 20 mg/L 40 mg/L, 60 mg/L 80 mg/L, 100 mg/L	Bai et al. (2014)
	Inhibition of alcohol-induced oxidative damage	L02 cells	10 μM, 20 µM 40 μM, 80 µM	Yao et al. (2015)
	Inhibits high glucose-induced oxidative damage	L02 cells	40 μM, 80 µM 160 µM	Liu et al. (2023)
Anti-inflammatory	Inhibition of LPS-stimulated secretion of proinflammatory cytokines	RAW264.7 cells	10 μM, 20 µM 40 µM	Yao et al. (2017)
	Inhibition of carrageene-induced plantar swelling in rats	Rats	1% (w/w)	Sridevi et al. (2017)
Membrane stabilizing	Reduces the permeability of the inner mitochondrial membrane to small molecule hydrophilic solutes	Inner Mitochondrial Membrane	1.5 mol%	Eriksson et al. (2018)
	To improve the membrane resistance to deformation and the resistance of *E.coli* to osmotic pressure	*Escherichia coli*	1.6 mol%	Eriksson et al. (2019)

## 2 Bioactivity of solanesol manuscript formatting

### 2.1 Antimicrobial activity

Currently, there is also literature that proves solanesol has antimicrobial effects. In 2007, Chen and colleagues utilized agar diffusion and twofold dilution methods to assess the antimicrobial activity of solanesol against a variety of standard bacterial strains (including *Staphylococcus aureus*, *Bacillus cereus*, *Escherichia coli*, and *Pseudomonas aeruginosa*) as well as clinically isolated strains (such as *Staphylococcus aureus*, *Bacillus subtilis*, *Mycobacterium phlei*, *Escherichia coli*, and *Pseudomonas aeruginosa*). The findings revealed that solanesol exhibited significant inhibitory effects against both Gram-negative bacteria (*E. coli* and *P. aeruginosa*) and Gram-positive bacteria (*S. aureus* and *M. phlei*), albeit showing weaker activity against *B. subtilis* and *B. cereus* (Chen et al., 2007). In 2008, Guo et al. utilized three extraction methods—supercritical extraction, Soxhlet extraction, and ultrasonic extraction—to isolate solanesol from tobacco leaves. The study found that solanesol, irrespective of the extraction method used, demonstrated the strongest antibacterial effect against *S. aureus*, followed by *B. subtilis*, with the least effectiveness observed against *E. coli* (Guo et al., 2008).

### 2.2 Antioxidant property

**FIGURE 2:**
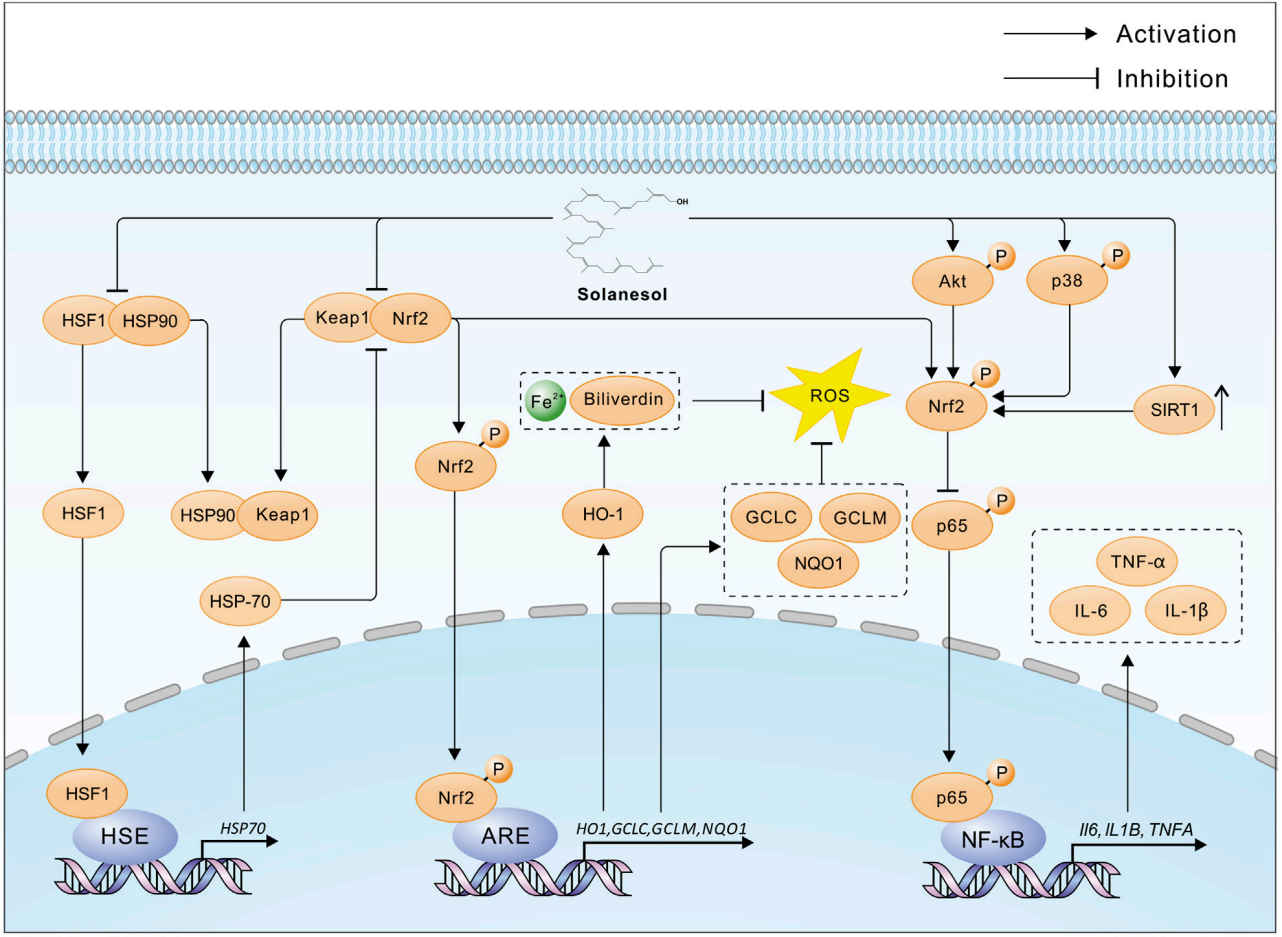
The multiple pharmacological mechanisms of solanesol. HSF1, Heat shock factor protein 1; HSP90, Heat shock protein 90; Keap1, Kelch-like ECH-associated protein 1; Nrf2, Nuclear factor erythroid 2-related factor 2; HSE, Heat shock element; ARE, Antioxidant response element; HO-1, Heme oxygenase 1; GCLC, Glutamate--cysteine ligase catalytic subunit; GCLM, Glutamate--cysteine ligase regulatory subunit; NQO1, NAD(P)H dehydrogenase [quinone] 1; ROS, Reactive oxygen species; HSP70, Heat shock protein 70; Akt, RAC-alpha serine/threonine-protein kinase; p38, Mitogen-activated protein kinase 14; SIRT1, Sirtuin 1; p65, RELA proto-oncogene, NF-kB subunit; IL-1β, Interleukin 1 beta; IL-6, Interleukin 6; TNF-α, tumor necrosis factor alpha; Solanesol disrupts the HSF1-HSP90 and Keap1-Nrf2 complexes, facilitating the liberation and nuclear translocation of Nrf2, which in turn initiates the transcription of ARE-mediated genes such as *HO1*, *GCLC*, *GCLM*, and *NQO1*. The enzymatic action of HO-1 results in an increased cytosolic concentration of Fe2+ and biliverdin, which suppresses the generation of ROS. Additionally, HSF1 promotes the transcription of HSP70, further enhancing the release of Nrf2 and bolstering the antioxidative defense. Furthermore, solanesol activates Nrf2 through the phosphorylation of Akt and p38 proteins, along with upregulating the expression of SIRT1, thereby inhibiting the phosphorylation of the p65 protein and subsequent transcription of pro-inflammatory cytokines such as *IL6*, *IL1B*, and *TNFA*, exerting an anti-inflammatory effect.

Solanesol, containing nine non-conjugated double bonds, has the capability to absorb free radicals (Qi et al., 2005). In 2008, Huang and colleagues utilized supercritical carbon dioxide with ethanol as a co-solvent to extract solanesol from tobacco, discovering that the antioxidant activity of the extracts was closely correlated with the yield of solanesol, with a determination coefficient of 0.946 (Huang et al., 2008). Further research by Ma and others revealed that solanesol possesses strong *in vitro* antioxidant activity, with its capacity to scavenge superoxide anions and hydroxyl radicals being comparable to that of the water-soluble vitamin E (Trolox), and it can inhibit microsomal lipid peroxidation (Ma et al., 2011). In 2014, Bai and team demonstrated that solanesol could effectively absorb ultraviolet radiation, scavenge lipid radicals, and inhibit tyrosinase activity, suggesting its potential in preventing skin aging and the formation of age spots (Bai et al., 2014). In 2015, Yao and colleagues found in an alcohol-induced L02 hepatocyte oxidative damage model that solanesol could disrupt the heat shock protein (Hsp) 90-heat shock factor 1 (HSF1) complex, promote the binding of Hsp90 to Keap1 (Kelch-like ECH-associated protein 1), thereby releasing nuclear factor-erythroid 2 related factor 2 (Nrf2). Nrf2 then enters the nucleus and binds to the antioxidant response element (ARE), initiating the transcription of downstream genes such as *H O -1*, *GCLC* and *GCLM* (Yao et al., 2015) (Figure 2). HO-1, a rate-limiting enzyme in heme catabolism, breaks down heme into biliverdin, iron, and carbon monoxide, exhibiting antioxidant effects (Mansouri et al., 2022). The enzyme complex formed by GCLC and GCLM, known as glutamate cysteine ligase (GCL), is the rate-limiting enzyme in the biosynthesis of glutathione (GSH), affecting intracellular GSH levels (Liu et al., 2022). GSH protects cells from oxidative stress induced by high levels of reactive oxygen species (ROS), maintaining the cellular redox state. Additionally, Solanesol disrupts the Hsp90-HSF1 complex, releasing HSF1 which then enters the nucleus and binds to the heat shock element (HSE). This initiates the transcription of downstream HSP70 genes, further activating the Keap1/Nrf2/ARE pathway and enhancing antioxidant effects (Yao et al., 2015). In 2023, Liu and team studied a high-glucose-induced L02 hepatocyte oxidative damage model and found that solanesol could decrease the expression of Keap1 and promote the translocation of Nrf2 to the nucleus, activating the Keap1/Nrf2/ARE pathway. This activation increases the expression of downstream *HO1* and *NQO1*, inhibits the expression and secretion of alanine aminotransferase (ALT), aspartate aminotransferase (AST), and lactate dehydrogenase (LDH), and improves the activities of superoxide dismutase (SOD), catalase (CAT), and glutathione peroxidase (GSH-Px). Thus, it balances intracellular ROS levels, inhibits lipid peroxidation of biomembranes, restores mitochondrial membrane potential, and protects L-02 cells under high-glucose conditions (Liu et al., 2023) (Figure 2).

### 2.3 Anti-inflammatory activity

The anti-inflammatory effects of solanesol were further explored by Yao et al., in 2017 within an LPS-stimulated macrophage model (RAW264.7 cells). The study illustrated that solanesol activates Nrf2 through phosphorylation of p38 and Akt, promoting Nrf2’s nuclear translocation and the initiation of *HO1* transcription. This process subsequently inhibited the secretion levels of IL-1β, IL-6, and TNF-α in LPS-stimulated RAW264.7 cells (Yao et al., 2017; Campbell et al., 2021) (Figure 2). Additionally, solanesol was shown to induce the expression of microtubule-associated protein 1 light chain 3B-II (LC3B-II) in a dose- and time-dependent manner, exerting its anti-inflammatory effect through the induction of autophagy. In the same year, Sridevi and colleagues demonstrated the significant *in vivo* anti-inflammatory effects of solanesol by preparing a solanesol gel and testing it in a rat paw edema experiment (Sridevi et al., 2017).

### 2.4 Membrane stabilization

In 2018, research by Eriksson et al. revealed that solanesol could mimic components of the inner mitochondrial membrane (IMM), integrating into liposomes to decrease the permeability of IMM to small hydrophilic solutes without affecting lipid stacking within the IMM (Eriksson et al., 2018). Another study further indicated that solanesol enhances the membrane’s resistance to deformation, thereby improving *E. coli’s* osmotic pressure resistance (Eriksson et al., 2019).

## 3 Pharmacological action of solanesol in diseases

### 3.1 Inflammatory diseases

**FIGURE 3:**
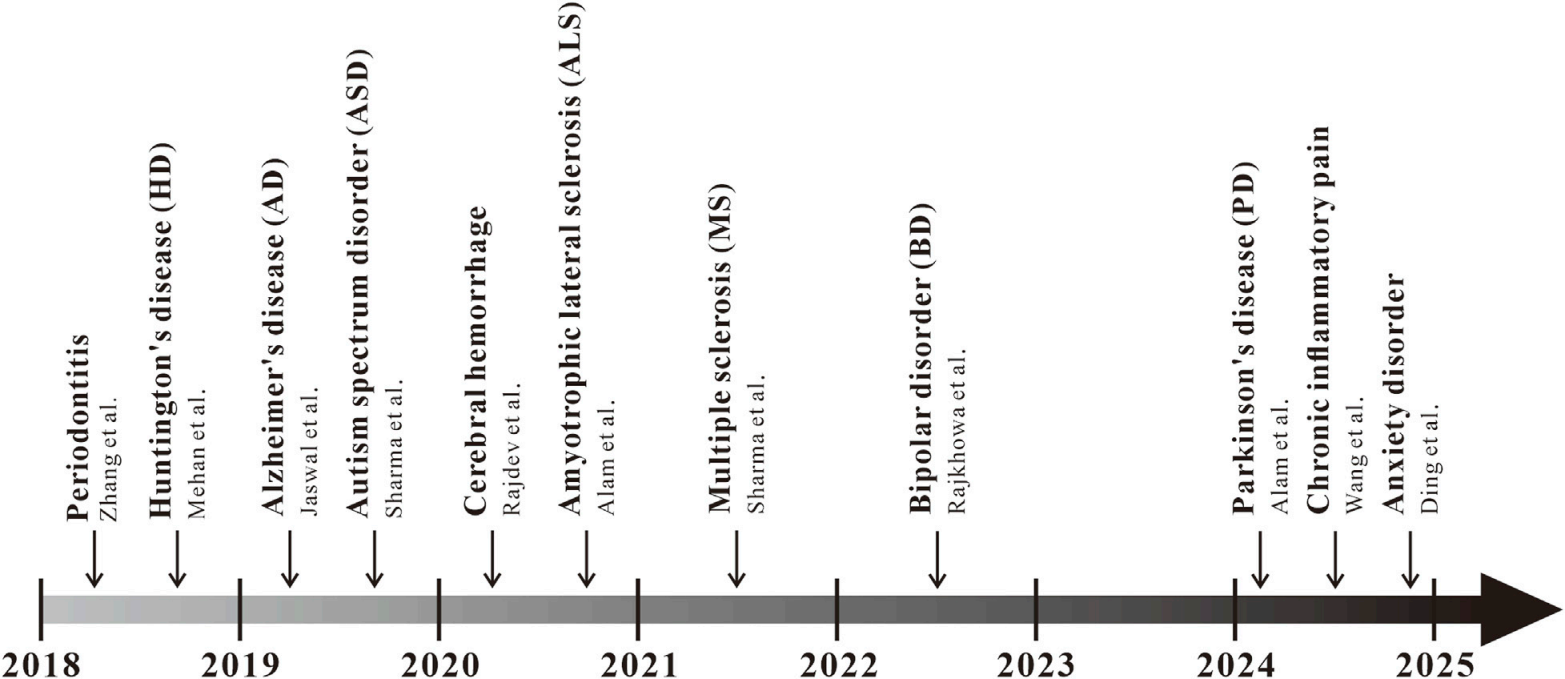
Progress of the pharmacological action of solanesol in diseases Starting from 2018, a multitude of studies have gradually focused on the pharmacological action of solanesol in diseases, such as periodontitis, Huntington’s disease (HD), Alzheimer’s disease (AD), autism spectrum disorder (ASD), cerebral hemorrhage, amyotrophic lateral sclerosis (ALS), multiple sclerosis (MS), bipolar disorder (BD), chronic inflammatory pain, and anxiety disorders.

In recent years, there has been a growing amount of research on solanesol in animal models of inflammatory diseases, such as periodontitis, chronic inflammatory pain, and anxiety induced by inflammation (Figure 3). In 2018, Zhang and colleagues conducted a study on the therapeutic effects of solanesol at three different concentrations—15 mg/kg, 30 mg/kg, and 60 mg/kg—on a rat model of experimental periodontitis (Zhang et al., 2018; Shang et al., 2023; Sczepanik et al., 2020). The research found that all three doses of solanesol significantly reduced the levels of IL-1β, TNF-α, and prostaglandin E2 (PGE2) in rat plasma. Additionally, solanesol increased the activity of superoxide dismutase (SOD) and glutathione peroxidase (GSH-Px), decreased malondialdehyde (MDA) content, improved systemic oxidative stress, alleviated alveolar bone loss, and mitigated periodontal inflammation. Intraplantar injection of complete Freund’s adjuvant (CFA) is a classic mouse model of anxiety-like behavior induced by neuroinflammation (Lazarević et al., 2024). Previously, a study found that a 50 mg/kg dose of solanesol could reverse mechanical allodynia and thermal hypersensitivity induced by CFA injection, alleviate anxiety-like syndromes associated with chronic inflammatory pain, and significantly reduce the levels of the inflammatory factors TNF-α and IL-1β in the mouse spinal cord. Immunohistochemical staining results further showed that the activation of microglia and astrocytes induced by CFA injection was significantly inhibited (Wang et al., 2024). After treating mice with 50 mg/kg solanesol for 1 week in a CFA-induced neuroinflammation mouse model, Ding et al. found a significant improvement in mouse anxiety behavior, reduced activation of microglia and astrocytes in the anterior cingulate cortex, downregulated TIA1, thereby lowering the levels of pro-inflammatory cytokines IL-1β and TNF-α. These studies reveal the potential of solanesol in treating inflammatory diseases, warranting further exploration of the mechanisms by which solanesol acts in inflammatory conditions, and providing direction for future pharmacological research on solanesol (Ding et al., 2024).

### 3.2 Central nervous system diseases

Over the past decade, a substantial body of research has focused on the neuroprotective effects of solanesol, demonstrating promising results in various animal disease models (Figure 3). In 2018, Mehan and colleagues explored solanesol’s neuroprotective effects against Huntington’s disease (HD), suggesting that solanesol may slow disease progression by improving mitochondrial dysfunction and reducing oxidative stress-induced neuronal damage (Jiang et al., 2023; Mehan et al., 2018a). Further research by Mehan and colleagues demonstrated that solanesol at doses of 5, 10, and 15 mg/kg could mitigate HD-like symptoms in rats, including memory impairments, reduced grip strength, postural abnormalities, and cognitive deficits induced by 3-nitropropionic acid, by activating coenzyme Q10 to repair mitochondrial damage. Significant improvements were observed in the histological alterations of the hippocampus, basal ganglia, and cerebral cortex in rats, along with alleviation of inflammation and oxidative damage in rat brain tissues (Mehan et al., 2018b). In 2019, Jaswal et al. created an Alzheimer’s disease (AD)-like rat model using intracerebroventricular injections of streptozotocin (Jaswal et al., 2019). They discovered that a 21-day combined treatment with solanesol (10 mg/kg) and epigallocatechin gallate (20 mg/kg) effectively reduced Ca2+ overload, lower levels of lipid peroxidation, superoxide dismutase (SOD), glutathione (GSH), and cytochrome C by closing the mitochondrial permeability transition pore, significantly improving the rats’ memory and learning capabilities. In the same year, Sharma from Mehan’s team utilized an intracerebroventricular injection of propionic acid to create an autism rat model, investigating solanesol’s neuroprotective effects (Hirota and King, 2023; Sharma et al., 2019). The study found that solanesol alone at doses of 40 and 60 mg/kg or in combination with drugs like aripiprazole, risperidone, clozapine, and donepezil could restore mitochondrial respiratory chain complex enzyme activities (complexes I, II, and V) and coenzyme Q10 activity, increase levels of neurotransmitters dopamine, acetylcholine, and glutamate, reduce levels of inflammation markers TNF-α and IL-1β, decrease oxidative stress markers acetylcholinesterase (AchE), GSH, and LDH, and improve cognitive deficits and biochemical markers in rats. Long-term treatment with solanesol and these drugs significantly improved long-term memory, depressive behavior, and muscle coordination in autistic rats, demonstrating its neuroprotective effects against autism.

In 2020, Alam from Mehan’s team investigated the therapeutic effects of solanesol alone at doses of 15 and 30 mg/kg and in combination with riluzole in a methylmercury-induced amyotrophic lateral sclerosis (ALS) rat model (Alam et al., 2020). The study found that long-term treatment elevated adenylate cyclase and mitochondrial coenzyme Q10 levels, inhibited inflammation and oxidative stress, reversed alterations in neurochemical substances, and improved grip strength and cognitive function in rats. Furthermore, Rajdev from Mehan’s team induced cerebral hemorrhage in rats by injecting autologous blood into the brain and found that long-term treatment with solanesol alone at doses of 40 and 60 mg/kg or in combination with drugs like donepezil, memantine, celecoxib, and pregabalin restored mitochondrial respiratory chain complex enzyme activities (complexes I, II, and V) and coenzyme Q10 levels in brain mitochondria (Sheth, 2022; Rajdev et al., 2020). This treatment improved levels of dopamine, glutamate, gamma-aminobutyric acid (GABA), and acetylcholine (Ach), reduced acetylcholinesterase (AchE) levels, suppressed the expression of neuroinflammatory cytokines (TNF-α, IL-6, IL-1β), and improved brain oxidative stress markers such as nitrate concentration, GSH, and SOD. It significantly ameliorated neuronal mitochondrial damage-associated post-hemorrhagic behavioral and neurochemical dysfunctions. In 2021, the team led by Mehan, with Sharma et al., investigated the effects of long-term treatment with solanesol at doses of 40 mg/kg and 80 mg/kg in a rat model of multiple sclerosis (MS) induced by ethidium bromide (Charabati et al., 2023; Sharma et al., 2021). Long-term treatment with both doses of solanesol was shown to upregulate the levels of SIRT-1 and myelin basic protein in rat brain tissues, restore the activities of mitochondrial respiratory chain complexes (complexes I, II, and V), improve neurotransmitter levels, inhibit the production of inflammatory cytokines TNF-α and IL-1β, significantly reduce oxidative stress levels, regulate apoptosis-related factors such as caspase-3, Bax, and Bcl-2, decrease the volume of brain demyelination, repair the pathological state of the rat brain, and improve motor functions, grip strength, memory, and cognitive abilities in rats, demonstrating significant neuroprotective effects. In 2022, Rajkhowa and colleagues conducted research on rats induced with bipolar disorder (BD)-like symptoms using ouabain (OUA) (Nierenberg et al., 2023; Rajkhowa et al., 2022). They discovered that solanesol, at doses of 40 mg/kg and 80 mg/kg, could increase the levels of SIRT-1 in cerebrospinal fluid, brain homogenate, and plasma samples. This increase led to improvements in the levels of apoptosis markers—caspase-3, Bax, and Bcl-2—restoration of mitochondrial respiratory chain complex enzymes (complexes I, II, IV, and V), reduction in the expression of inflammatory cytokines TNF-α and IL-1β, elevation of serotonin and acetylcholine levels, decrease in dopamine and glutamate levels, reduction of oxidative stress, and restoration of altered Na/K ATPase levels. Additionally, the combination of solanesol at a dose of 80 mg/kg with lithium significantly reduced brain neuroglial cell proliferation, restored neuronal cell structures, decreased cell apoptosis, and alleviated manic-like and depressive-like behaviors in BD model rats.

In 2024, research has found that in a zebrafish Parkinson’s disease (PD) model induced by Tramadol, motor dysfunction and motor coordination in the zebrafish PD model were reversed after treatment with solanesol alone at doses of 25, 50, and 100 mg/kg, as well as in combination with standard drugs (Morris et al., 2024; Alam et al., 2024). Following treatment with solanesol, the levels of dopamine and norepinephrine in the brains of zebrafish were restored, serotonin levels were increased, GABA levels were improved, the neurotransmitter system was repaired, levels of TNF-α and IL-1β were reduced, and levels of IL-10 were increased. Additionally, solanesol significantly reduced the levels of oxidative stress induced by Tramadol, improved lipid peroxidation, decreased the concentration of malondialdehyde (MDA), and restored the levels of reduced glutathione (GSH) and superoxide dismutase (SOD). Histopathological results showed that after treatment with solanesol at various concentrations, neuronal cell damage and neuronal cell density were both improved in a dose-dependent manner.

## 4 Druggability

Solanesol, a long-chain unsaturated fatty alcohol composed of nine isoprene units with nine non-conjugated double bonds, exhibits unique chemical properties that underpin its diverse bioactivities. Its low polarity and insolubility in water, coupled with solubility in organic solvents like hexane and ethanol, facilitate its integration into lipid membranes and nanocarrier systems. The extended hydrophobic chain and conjugated double-bond system contribute to its strong antioxidant capacity by scavenging free radicals and stabilizing lipid peroxidation. Furthermore, the structural flexibility of solanesol enables interactions with key molecular targets. Despite significant progress in elucidating solanesol’s bioactivities, current research gaps remain. The precise molecular targets and downstream signaling pathways of solanesol in various disease models remain incompletely characterized, necessitating advanced techniques such as artificial intelligence (AI) and machine learning-based target prediction, activity-based protein profiling, or molecular docking to identify binding partners. Additionally, the structure-activity relationship (SAR) of solanesol, particularly how specific structural motifs (e.g., double-bond configuration, chain length) influence its pharmacological efficacy, requires systematic exploration.

Future research should prioritize translating preclinical findings into clinical applications. While animal studies demonstrate solanesol’s therapeutic potential in neurodegenerative and inflammatory diseases, rigorous pharmacokinetic studies and human trials are essential to validate its safety and efficacy. Emerging applications in nanotechnology also hold promise. Solanesol’s amphiphilic nature and compatibility with lipid-based systems position it as an ideal candidate for advanced drug delivery platforms, including liposomes or polymeric nanoparticles, to improve drug solubility and targeted delivery. Collaborative efforts integrating computational biology, structural biology, and synthetic chemistry will be pivotal in unlocking solanesol’s full medicinal potential. By addressing these challenges and opportunities, solanesol could emerge as a cornerstone in the development of next-generation therapeutics for complex diseases, bridging the gap between natural product discovery and clinical innovation.

## 5 Conclusion

So far, solanesol has been shown to disrupt the HSF1-HSP90 and Keap1-Nrf2 complexes, phosphorylate Akt and p38, and thereby facilitate the release and nuclear translocation of Nrf2. This activation initiates the transcription of downstream genes such as *HO1*, increasing the cytoplasmic levels of Fe^2+^ and biliverdin, and inhibiting the production of ROS. Additionally, solanesol enhances the activity of mitochondrial respiratory chain complexes and improves neurotransmitter levels, inhibiting the secretion of inflammatory cytokines. Solanesol, apart from exhibiting antibacterial, antioxidant, anti-inflammatory, and membrane-stabilizing effects, has also shown promising therapeutic potential in animal models of inflammatory diseases and central nervous system disorders. Therefore, as a natural product, solanesol is worth further exploration. Although significant progress has been made in the pharmacological research of solanesol, there are still shortcomings. Current studies are limited to the pharmacological effects of solanesol and some superficial mechanistic explorations, without clarifying the specific targets of solanesol’s action. The exploration of signaling pathways is also not comprehensive or in-depth. Moreover, existing animal experiments have only explored the role of solanesol in central nervous system diseases, with only partial validation in cell experiments for other diseases. Therefore, the purpose of this review is to summarize the latest research progress of solanesol, providing ideas for subsequent research on the pharmacological effects of solanesol.

## 6 Data sources

This review systematically compiled studies on solanesol through searches in major databases, including NCBI (National Center for Biotechnology Information, https://pubmed.ncbi.nlm.nih.gov), CNKI (China National Knowledge Infrastructure, https://www.cnki.net/), and Elsevier (https://www.elsevier.cn/), among others. The search terms included “solanesol,” “nonaprenol alcohol,” “antimicrobial,” “antioxidant,” “anti-inflammatory,” “neuroprotective,” and “pharmacological mechanisms.” Inclusion criteria encompassed peer-reviewed articles in English or Chinese focusing on solanesol’s bioactivity, pharmacological effects, or mechanisms. Exclusion criteria included non-original research (e.g., reviews, conference abstracts) and studies without experimental validation.
